# Supplementary material for: Characterizing the interactions between influenza and respiratory syncytial viruses and their implications for epidemic control
Source: Nat Commun. 2024 Nov 20;15:10066. doi: 10.1038/s41467-024-53872-4 (PMC11579344; doi:10.1038/s41467-024-53872-4)
Supplement: Supplementary file 2 — Reporting Summary [file 41467_2024_53872_MOESM2_ESM.pdf]

nature portfolio

Corresponding author(s): Kramer, SC

Last updated by author(s): May 10, 2024

Reporting Summary

Nature Portfolio wishes to improve the reproducibility of the work that we publish. This form provides structure for consistency and transparency in reporting. For further information on Nature Portfolio policies, see our Editorial Policies and the Editorial Policy Checklist.

Statistics

For all statistical analyses, confirm that the following items are present in the figure legend, table legend, main text, or Methods section.

n/a	Confirmed	
☐	☒	The exact sample size (*n*) for each experimental group/condition, given as a discrete number and unit of measurement
☒	☐	A statement on whether measurements were taken from distinct samples or whether the same sample was measured repeatedly
☐	☒	The statistical test(s) used AND whether they are one- or two-sided<br>*Only common tests should be described solely by name; describe more complex techniques in the Methods section.*
☒	☐	A description of all covariates tested
☒	☐	A description of any assumptions or corrections, such as tests of normality and adjustment for multiple comparisons
☐	☒	A full description of the statistical parameters including central tendency (e.g. means) or other basic estimates (e.g. regression coefficient) AND variation (e.g. standard deviation) or associated estimates of uncertainty (e.g. confidence intervals)
☐	☒	For null hypothesis testing, the test statistic (e.g. *F*, *t*, *r*) with confidence intervals, effect sizes, degrees of freedom and *P* value noted<br>*Give P values as exact values whenever suitable.*
☒	☐	For Bayesian analysis, information on the choice of priors and Markov chain Monte Carlo settings
☒	☐	For hierarchical and complex designs, identification of the appropriate level for tests and full reporting of outcomes
☐	☒	Estimates of effect sizes (e.g. Cohen's *d*, Pearson's *r*), indicating how they were calculated

Our web collection on statistics for biologists contains articles on many of the points above.

Software and code

Policy information about availability of computer code

Data collection	NA
Data analysis	All analyses were conducted in R version 4.2.3. The model was coded and run using the package pomp (version 5.4), and fit using the sbplx algorithm via the package nloptr (version 2.0.3). All code can be found at: https://github.com/sarahckramer/resp_virus_interactions

For manuscripts utilizing custom algorithms or software that are central to the research but not yet described in published literature, software must be made available to editors and reviewers. We strongly encourage code deposition in a community repository (e.g. GitHub). See the Nature Portfolio guidelines for submitting code & software for further information.

Data

Policy information about availability of data

All manuscripts must include a data availability statement. This statement should provide the following information, where applicable:

- Accession codes, unique identifiers, or web links for publicly available datasets
- A description of any restrictions on data availability
- For clinical datasets or third party data, please ensure that the statement adheres to our policy

Links to all publicly-available data used in this study can be found, along with the code used for the analyses, at: https://github.com/sarahckramer/resp_virus_interactions

Research involving human participants, their data, or biological material

Policy information about studies with human participants or human data. See also policy information about sex, gender (identity/presentation), and sexual orientation and race, ethnicity and racism.

Reporting on sex and gender	NA
Reporting on race, ethnicity, or other socially relevant groupings	NA
Population characteristics	NA
Recruitment	NA
Ethics oversight	NA

Note that full information on the approval of the study protocol must also be provided in the manuscript.

Field-specific reporting

Please select the one below that is the best fit for your research. If you are not sure, read the appropriate sections before making your selection.

☐ Life sciences ☐ Behavioural & social sciences ☒ Ecological, evolutionary & environmental sciences

For a reference copy of the document with all sections, see nature.com/documents/nr-reporting-summary-flat.pdf

Ecological, evolutionary & environmental sciences study design

All studies must disclose on these points even when the disclosure is negative.

Study description	We fit mathematical models of influenza and respiratory syncytial virus cocirculation to observed data from Hong Kong and Canada, in order to estimate the strength and duration of the interaction effect between the two viruses. There were no covariates to control for in this study.
Research sample	Publicly-available, weekly data on 1) the total number of tests performed for influenza and RSV, 2) the number of tests positive for influenza or RSV, and 3) the proportion of all-cause consultations due to influenza-like illness were obtained from the Centre for Health Protection of the Government of the Hong Kong Special Administrative Region and the Public Health Agency of Canada. These data were chosen for their availability, and because both datasets contain several years of data on both viruses.
Sampling strategy	As previously-collected data were used for this study, we did not conduct any sampling.
Data collection	Patients in public hospitals (Hong Kong), or in either hospitals or emergency departments (Canada) were tested for influenza and/or RSV if diagnostic information could potentially aid treatment. The number of patients with influenza-like illness were reported by outpatient clinics (Hong Kong) and primary care providers (Canada).
Timing and spatial scale	Relevant data in Hong Kong cover the period from January 2014 through mid-February 2020; data in Canada cover from September 2010 through August 2014. In both locations, data were available at a weekly time scale, and were collected from patients from throughout the country.
Data exclusions	We did not consider data collected during the 2009 influenza pandemic or during the recent pandemic of SARS-CoV-2. This is because typical circulation patterns of both influenza and RSV were strongly affected by both pandemics.
Reproducibility	All code to repeat the analyses has been made publicly available.
Randomization	Models were fit to the data described above; no randomization was conducted.
Blinding	This is a modeling study, in which a model was fit to data in order to estimate several key parameters of interest; blinding is not relevant to this study design.

Did the study involve field work? ☐ Yes ☒ No

Reporting for specific materials, systems and methods

We require information from authors about some types of materials, experimental systems and methods used in many studies. Here, indicate whether each material, system or method listed is relevant to your study. If you are not sure if a list item applies to your research, read the appropriate section before selecting a response.

Materials & experimental systems

n/a	Involved in the study
☒	☐ Antibodies
☒	☐ Eukaryotic cell lines
☒	☐ Palaeontology and archaeology
☒	☐ Animals and other organisms
☒	☐ Clinical data
☒	☐ Dual use research of concern
☒	☐ Plants

Methods

n/a	Involved in the study
☒	☐ ChIP-seq
☒	☐ Flow cytometry
☒	☐ MRI-based neuroimaging

Plants

Seed stocks	NA
Novel plant genotypes	NA
Authentication	NA
